# Dynamic chiral magnetic effect and anisotropic natural optical activity of tilted Weyl semimetals

**DOI:** 10.1038/s41598-020-59385-6

**Published:** 2020-02-14

**Authors:** Urmimala Dey, S. Nandy, A. Taraphder

**Affiliations:** 1 Centre for Theoretical Studies, Indian Institute of Technology Kharagpur, Kharagpur, 721302 India; 2 Department of Physics, University of Virginia, Charlottesville, VA 22904 USA; 3 Department of Physics, Indian Institute of Technology Kharagpur, Kharagpur, 721302 India

**Keywords:** Mathematics and computing, Physics

## Abstract

We study the dynamic chiral magnetic conductivity (DCMC) and natural optical activity in an inversion-broken tilted Weyl semimetal (WSM). Starting from the Kubo formula, we derive the analytical expressions for the DCMC for two different directions of the incident electromagnetic wave. We show that the angle of rotation of the plane of polarization of the transmitted wave exhibits remarkable anisotropy and is larger along the tilt direction. This striking anisotropy of DCMC results in anisotropic optical activity and rotary power, which can be experimentally observed as a topological magneto-electric effect of inversion-broken tilted WSMs. Finally, using the low energy Hamiltonian, we show that the DCMC follows the universal $\frac{{\bf{1}}}{{{\boldsymbol{\omega }}}^{{\bf{2}}}}$ decay in the high frequency regime. In the low frequency regime, however, the DCMC shows sharp peaks at the tilt dependent effective chemical potentials of the left-handed and right-handed Weyl points. This can serve as a signature to distinguish between the type-I and type-II Weyl semimetals.

## Introduction

The Weyl equations of high energy physics^1^ describe the emergent, linearly dispersing, low energy excitations of condensed matter systems known as Weyl semimetals (WSM)^2–9^. In these systems, which violate spatial inversion (SI) and/or time reversal (TR) symmetry^6–9^, two non-degenerate bands touch at isolated points in the momentum space which act as the source and sink of Abelian Berry curvature. The sources and sinks of Berry curvature define the Weyl points of opposite chirality, which come in pairs due to a no-go theorem by Nielsen and Ninomiya^10,11^. The nontrivial distribution of Berry curvature in WSMs lead to many anomalous transport properties in presence as well as in absence of external electric and magnetic fields such as Planar Hall effect and negative longitudinal magnetoresistance (LMR) due to the chiral or Adler-Bell-Jackiw anomaly^7–24^ and large anomalous Hall effect^25,26^. A particularly intriguing effect results when the Weyl points with opposite chirality occur at different energies in an inversion-broken Weyl semimetal, with the separation in energy called a chiral chemical potential. In this case, the system supports a charge current in response to an applied magnetic field even in the absence of an electric field. This effect, known as chiral magnetic effect (CME)^11,27–30^ in WSMs^12,16,19,31–34^, vanishes in the static limit^33^, and in equilibrium systems the current induced by a time-independent magnetic field is zero.

The anomalous Hall conductivity is identically zero for an inversion-broken Weyl semimetal with unbroken TRS, which is in contrast to a TRS-broken WSM. It can be characterized by the presence of a non-zero chiral chemical potential. A non-zero *dynamic* chiral magnetic effect (DCME) may appear as a consequence of the non-vanishing chiral chemical potential in an inversion-broken WSM, which is given by

1 $${\bf{j}}({\bf{q}},\omega )={\sigma }^{chiral}({\bf{q}},\omega ){\bf{B}}({\bf{q}},\omega )$$

here, *σ*^*chiral*^(**q**, *ω*) represents dynamic chiral magnetic conductivity (DCMC), non-zero for finite *omega* only. Goswami *et al*^19^. had shown that the natural optical activity of an SI-broken Weyl semimetal is related to DCMC. This phenomenon, also known as optical gyrotropy^35,36^, may be used as a signature of the topological magnetoelectric effect of a WSM that breaks SI symmetry. Together with Maxwell relation **B** = **q** × **E**/*ω* (for *c* = 1), Eq. (1) leads to a full charge conductivity tensor

2 $${\sigma }_{\alpha \beta }({\bf{q}},\omega )=-\,\frac{{\sigma }^{chiral}({\bf{q}},\omega )}{\omega }{\epsilon }_{\alpha \beta \gamma }{q}_{\gamma },$$

where $\epsilon$_*αβγ*_ is the fully anti-symmetric Levi-Civita tensor. The natural optical activity of an inversion-broken system is obtained from the *q*-linear part of the full conductivity tensor *σ*_*αβ*_(**q**, *ω*) in presence of TRS. It follows then that a non-zero DCMC directly produces a non-zero gyrotropy and natural optical activity in spatial inversion-broken Weyl semimetals. This indeed can be measured experimentally.

In a TRS-broken system, the optical Hall conductivity causes the rotation of the plane of polarization of the transmitted light^37^. While in contrast, in an inversion-broken WSM, the rotation of polarization occurs due to non-zero gyrotropic current i.e., the consequent natural optical activity can be measured experimentally. In this paper, we calculate the dynamic chiral magnetic conductivity of an inversion-symmetry-breaking tilted Weyl semimetal and angles of rotation of the plane of polarization of the transmitted light for different incident electromagnetic wave directions, using the relation between DCMC and the rotary power.

Apart from the conventional type-I WSMs, another type of Weyl semimetals (type-II) have been theoretically proposed^38^ on the basis of Fermi surface topology and symmetry considerations and are experimentally realised in several realistic materials^39,40^. In the case of a type-I WSM, two bands cross linearly at the Fermi level with vanishing density of states, whereas, a type-II WSM produces finite electron and hole pockets at the Fermi level as a result of finite tilting of the energy spectra^38,39^. A type-II Weyl fermion breaks the Lorentz invariance explicitly and does not have a high energy analogue; however, it can appear as a low energy excitation in condensed matter systems because of the absence of Lorentz symmetry^39^. The type-II WSMs can be distinguished from the conventional type-I WSMs by observing their magneto-optical response in presence of an external magnetic field^41,42^ or using artificial gravitational effect induced by lattice strain^43^. Presence of finite density of states at the Fermi level and tilted energy spectra in type-II WSMs give rise to many interesting novel phenomena such as chiral anomaly-induced anisotropic LMR^44^ and anisotropic planar Hall effect^45^. In this work, we calculate the dynamic chiral magnetic conductivity in the framework of Kubo formalism. Based on an inversion-symmetry-broken lattice model with chiral chemical potential, we show that the DCMC and the resultant natural optical activity and rotary power in type-II WSMs are finite and show strong anisotropy in space. Therefore, this anisotropic topological magneto-electric effect can serve as a characteristic feature of inversion-broken tilted WSMs. Moreover, constructing a continuum model from the lattice Hamiltonian, we show the frequency dependence of the real part of the DCMC. In the high frequency limit, the dynamical chiral magnetic conductivity is found to follow the universal $\frac{1}{{\omega }^{2}}$ decay, whereas in the low frequency regime, the DCMC shows sharp peaks at the tilt dependent effective chemical potentials of the left and right-handed Weyl points, allowing one to distinguish between the type-I and type-II Weyl semimetals.

In the second section, we introduce an inversion-symmetry-broken lattice Hamiltonian which produces tilted Weyl points with chiral chemical potential. Section three describes the formalism for the Berry curvature-induced DCMC. The expressions for DCMC are calculated for two different directions of the incident electromagnetic wave. The calculation of rotary power is presented in section four. In section five, we construct a continuum model and show the frequency dependence of the real part of the DCMC. Finally in section six, we summarize the results and draw conclusions thereof.

## Lattice Hamiltonian for Inversion-Symmetry-Broken Tilted Weyl Semimetal

We adopt a two-band model defined on a cubic lattice, which reproduces all the topological aspects of an inversion-broken tilted Weyl semimetal. We consider the Hamiltonian

3 $$\begin{array}{ccc}{\mathcal{H}}({\bf{k}}) & = & {t}_{2}[\cos ({k}_{x}+{k}_{y})+\delta \ \cos ({k}_{x}-{k}_{y})]{\sigma }_{0}+{t}_{1}[(\cos {k}_{0}-\cos {k}_{x})+\delta (1-\cos {k}_{z})]{\sigma }_{z}\\  &  & +{t}_{1}[(\cos {k}_{0}-\cos {k}_{y})+\delta (1-\cos {k}_{z})]{\sigma }_{x}+{t}_{1}\ \sin {k}_{z}{\sigma }_{y}\\  & = & \sum _{{\bf{k}}}{{\mathcal{N}}}_{0,{\bf{k}}}{\sigma }_{0}+{\boldsymbol{\mathcal{N}}}_{{\bf{k}}}\cdot {\boldsymbol{\sigma }}\end{array}$$

where, *t*_1_ and *t*_2_ are the hopping parameters, *δ* (≠ 1) is a constant, *σ*’s are the Pauli spin matrices and ${{\mathcal{N}}}_{0,{\bf{k}}}$ and ${\boldsymbol{\mathcal{N}}}_{{\bf{k}}}$ are given by

$${{\mathcal{N}}}_{0,{\bf{k}}}={t}_{2}[\cos ({k}_{x}+{k}_{y})+\delta \cos ({k}_{x}-{k}_{y})]$$

4 $${\boldsymbol{\mathcal{N}}}_{{\bf{k}}}=\{{t}_{1}[(\cos {k}_{0}-\cos {k}_{y})+\delta (1-\cos {k}_{z})],{t}_{1}\sin {k}_{z},{t}_{1}[(\cos {k}_{0}-\cos {k}_{x})+\delta (1-\cos {k}_{z})]\}$$

the energy eigenvalues of ${\mathcal{H}}({\bf{k}})$ are

5 $${{\mathcal{E}}}_{l,{\bf{k}}}={{\mathcal{N}}}_{0,{\bf{k}}}+{(-1)}^{l}\left|{\boldsymbol{\mathcal{N}}}_{{\bf{k}}}\right|$$

where, *l* (=1, 2) is the band index.

For *t*_2_ = 0 and *δ* > 1, four gapless points arise in the *k*_*z*_ = 0 plane at (*k*_0_, *k*_0_, 0), (*k*_0_, −*k*_0_, 0), (−*k*_0_, *k*_0_, 0) and (−*k*_0_, −*k*_0_, 0) and without any loss of generality we can consider $0 < {k}_{0} < \frac{\pi }{2}$. The right-handed Weyl points are located at ± (*k*_0_, *k*_0_, 0) and the left-handed Weyl points are located at ± (*k*_0_, − *k*_0_, 0). If *δ* is tuned to less than one, we get four more touching points at *k*_*z*_ = *π* plane. When *t*_2_ ≠ 0, the first term in Eq. (3) causes shifts in energies of the Weyl points of opposite chiralities. The right and the left-handed Weyl points now appear at *E*_*R*_ = ${t}_{2}[\cos (2{k}_{0})+\delta ]$ and *E*_*L*_ = ${t}_{2}[1+\delta \cos (2{k}_{0})]$ respectively, producing a constant chiral chemical potential *μ*_*ch*_ = (*E*_*R*_ − *E*_*L*_)/2 = ${t}_{2}(\delta -1){\sin }^{2}{k}_{0}$, which is essential for a non-zero DCMC.

**Figure 1:**
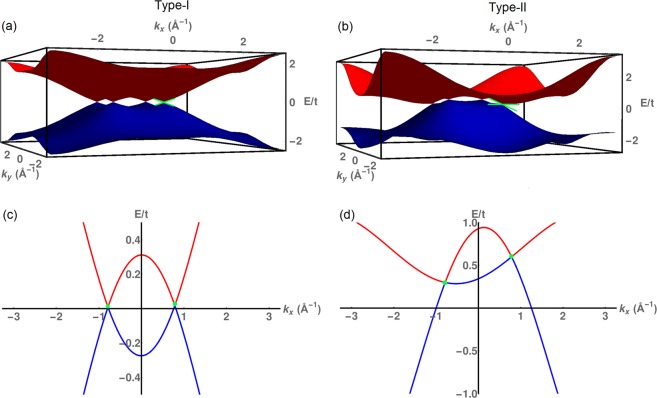
The energy dispersions of the lattice Hamiltonian for *k*_*z*_ = 0 with parameters *k*_0_ = $\frac{\pi }{4}$ and *δ* = 2, for different values of the ratio *r* = $\frac{{t}_{2}}{{t}_{1}}$. (**a**) Type-I WSM with *t*_2_ = 0.009*t* and *t*_1_ = *t*, (**b**) type-II WSM with *t*_2_ = 0.3*t* and *t*_1_ = *t*. Cuts through the Weyl points at *k*_*z*_ = 0 and *k*_*y*_ = $\frac{\pi }{4}$ (**c**) for type-I and (**d**) for type-II WSM using the same parameter values as in (**a**,**b**). When *r* is less than the critical value (*r*_*c*_), two non-degenerate bands meet at four type-I Weyl points. As *r* increases, the WPs start to tilt in the *x*-direction. When *r* is above *r*_*c*_ = 0.01, the Weyl nodes tilt further and two pairs of type-II WPs are observed. The green dots indicate WPs. Thus by tuning the ratio *r*, one can go from a type-I to a type-II model.

In Fig. 1, we have shown the energy spectrum of the Hamiltonian in Eq. (3) for different values of the ratio $r=\frac{{t}_{2}}{{t}_{1}}$. We see that when *r* is less than the critical value (*r*_*c*_), two bands meet at four type-I Weyl points. As *r* is increased, the Weyl points start to tilt in the *x*-direction. As *r* increases beyond *r*_*c*_, the Weyl nodes tilt further and we get two pairs of type-II Weyl points. Thus, by tuning the ratio $\frac{{t}_{2}}{{t}_{1}}$, we can go from a type-I to a type-II model.

For complete description of electron-dynamics in topological semimetals, we need to consider the effect of Berry curvature of Bloch bands, which acts as a magnetic field in the momentum space^46^. If |*u* > is the periodic amplitude of the Bloch wavefunction, then the Berry curvature of Bloch bands is defined as Ω(**k**) = ∇_**k**_ × < *u*|*i*∇_**k**_|*u* >. For a system preserving time-reversal symmetry, Ω(−**k**) = −Ω(**k**) and for a spatial inversion symmetric system, Ω(−**k**) = Ω(**k**). Therefore, Berry curvature acquires a non-zero value only when the system breaks either time-reversal symmetry or spatial inversion symmetry or both.

For the *l*-th Bloch band, Berry curvature is given by

6 $${\Omega }_{l,\alpha }({\bf{k}})={(-1)}^{l+1}{\Omega }_{\alpha }=\frac{{\epsilon }_{\alpha \beta \gamma }}{4{\left|{\boldsymbol{\mathcal{N}}}_{{\bf{k}}}\right|}^{3}}{\boldsymbol{\mathcal{N}}}_{{\bf{k}}}\cdot ({\partial }_{\beta }{\boldsymbol{\mathcal{N}}}_{{\bf{k}}}\times {\partial }_{\gamma }{\boldsymbol{\mathcal{N}}}_{{\bf{k}}})$$

with *α*, *β*, *γ* = *x*, *y*, *z*. Using Eqs. (4) and (6), the components of Berry curvature can be calculated as

$${\Omega }_{x}({\bf{k}})=\frac{1}{{2\left|{\boldsymbol{\mathcal{N}}}_{{\bf{k}}}\right|}^{3}}({{\mathcal{N}}}_{z,{\bf{k}}}{\partial }_{y}{{\mathcal{N}}}_{x}{\partial }_{z}{{\mathcal{N}}}_{y}-{{\mathcal{N}}}_{y,{\bf{k}}}{\partial }_{y}{{\mathcal{N}}}_{x}{\partial }_{z}{{\mathcal{N}}}_{z})$$

$${\Omega }_{y}({\bf{k}})=\frac{1}{{2\left|{\boldsymbol{\mathcal{N}}}_{{\bf{k}}}\right|}^{3}}({{\mathcal{N}}}_{x,{\bf{k}}}{\partial }_{x}{{\mathcal{N}}}_{z}{\partial }_{z}{{\mathcal{N}}}_{y}-{{\mathcal{N}}}_{y,{\bf{k}}}{\partial }_{x}{{\mathcal{N}}}_{z}{\partial }_{z}{{\mathcal{N}}}_{x})$$

7 $${\Omega }_{z}({\bf{k}})=\frac{1}{{2\left|{\boldsymbol{\mathcal{N}}}_{{\bf{k}}}\right|}^{3}}{{\mathcal{N}}}_{y,{\bf{k}}}{\partial }_{x}{{\mathcal{N}}}_{z}{\partial }_{y}{{\mathcal{N}}}_{x}$$

where, ${{\mathcal{N}}}_{\alpha ,{\bf{k}}}$ is the *α*-th component of ${\boldsymbol{\mathcal{N}}}_{{\bf{k}}}$ and ${\partial }_{\beta }{{\mathcal{N}}}_{\alpha }\equiv {\partial }_{{k}_{\beta }}{{\mathcal{N}}}_{\alpha }$ with *α*, *β* = *x*, *y*, *z*.

## Dynamic Chiral Magnetic Conductivity in the Lattice Model

Using Quantum Field theory calculations, it is possible to show that if a system breaks both SI and time reversal symmetries (TRS), it can show a magnetoelectric coupling^47^. In an SI broken system, the time derivative of the magnetoelectric coupling gives rise to a current in the direction of the applied field. This is known as the dynamic chiral magnetic current. If the scattering effects are negligible, the Kubo formula can be used to calculate the chiral magnetic conductivity:

8 $${\sigma }_{\gamma }^{chiral}({\bf{q}},\omega )=\frac{{\epsilon }_{\alpha \beta \gamma }}{2i{q}_{\gamma }}{\Lambda }_{\alpha \beta }({\bf{q}},i{\omega }_{m}\to \omega +i\delta )$$

where, **q** and *ω* are the wavevector and the frequency of the electromagnetic wave such that *ω* ≫ the scattering rate due to impurities and *α*, *β*, *γ* = *x*, *y*, *z*.

The current-current correlation function Λ_*αβ*_ can be expressed in terms of the fermionic and bosonic propagators ${\mathcal{G}}(i{\omega }_{n},{\bf{k}})$ and ${\mathcal{G}}(i{\omega }_{n}+i{\omega }_{m},{\bf{k}})$ as

9 $${\Lambda }_{\alpha \beta }=\frac{1}{\beta }\sum _{n}{\int }_{{\bf{k}}}\,{\rm{Tr}}\,\left[{j}_{\alpha }({\bf{k}}){\mathcal{G}}(i{\omega }_{n},{\bf{k}}+\frac{{\bf{q}}}{2})\times {j}_{\beta }({\bf{k}}){\mathcal{G}}(i{\omega }_{n}+i{\omega }_{m},{\bf{k}}-\frac{{\bf{q}}}{2})\right]$$

here, *ω*_*n*_ = (2*n* + 1)*π*T and *ω*_*m*_ = 2*mπ*T are the Matsubara frequencies for fermions and bosons respectively. *j*_*α*_(**k**)’s (*α* = *x*, *y*, *z*) are the current density operators which can be obtained from the Hamiltonian in presence of a gauge field **A**:

10 $${j}_{\alpha }({\bf{k}})={\left.\frac{\partial }{\partial {A}_{\alpha }}H({\bf{k}}-e{\bf{A}})\right|}_{{A}_{\alpha }=0}$$

for the lattice Hamiltonian in Eq. (3), the current density operators take the form:

11 $${j}_{\alpha }({\bf{k}})=\,-\,e\left[{\partial }_{\alpha }{{\mathcal{N}}}_{0,{\bf{k}}}+{\partial }_{\alpha }{\boldsymbol{\mathcal{N}}}_{{\bf{k}}}\cdot {\boldsymbol{\sigma }}\right]$$

and the propagator can be written as

12 $${\mathcal{G}}(i{\omega }_{n},{\bf{k}})=\frac{i{\omega }_{n}+\mu -{{\mathcal{N}}}_{0,{\bf{k}}}+{\boldsymbol{\mathcal{N}}}_{{\bf{k}}}\cdot {\boldsymbol{\sigma }}}{{(i{\omega }_{n}+\mu -{{\mathcal{N}}}_{0,{\bf{k}}})}^{2}-{\left|{\boldsymbol{\mathcal{N}}}_{{\bf{k}}}\right|}^{2}}.$$

In the following sections we calculate the dynamic chiral magnetic conductivity for two different directions of the incident electromagnetic wave.

### DCMC perpendicular to the tilt in the spectrum

We first consider the electromagnetic wave has only the *z*-component of the wavevector **q** i.e., **q** = (0, 0, q). So, it is relevant to calculate the *xy* and *yx*-components of Λ_*αβ*_ in this case. Writing Λ_*αβ*_ as

13 $${\Lambda }_{\alpha \beta }^{(z)}=\frac{{N}_{\alpha \beta }^{(z)}}{{D}_{\alpha \beta }^{(z)}}$$

and performing the trace in Eq. (9), we get the numerator (${N}_{\alpha \beta }^{(z)}$) as

14 $$\begin{array}{rcl}{N}_{\alpha \beta }^{(z)} & = & -4i{e}^{2}\left[{\partial }_{x}\,{{\mathcal{N}}}_{z}{\partial }_{y}\,{{\mathcal{N}}}_{0}\times \left({{\mathcal{N}}}_{y,{\bf{k}}+\frac{{\bf{q}}}{2}}\,{{\mathcal{N}}}_{x,{\bf{k}}-\frac{{\bf{q}}}{2}}-{{\mathcal{N}}}_{x,{\bf{k}}+\frac{{\bf{q}}}{2}}\,{{\mathcal{N}}}_{y,{\bf{k}}-\frac{{\bf{q}}}{2}}\right)\right.\\  &  & +{\partial }_{x}\,{{\mathcal{N}}}_{0}{\partial }_{y}\,{{\mathcal{N}}}_{x}\left({{\mathcal{N}}}_{y,{\bf{k}}+\frac{{\bf{q}}}{2}}\,{{\mathcal{N}}}_{z,{\bf{k}}-\frac{{\bf{q}}}{2}}-{{\mathcal{N}}}_{z,{\bf{k}}+\frac{{\bf{q}}}{2}}\,{{\mathcal{N}}}_{y,{\bf{k}}-\frac{{\bf{q}}}{2}}\right)\\  &  & -{\partial }_{x}\,{{\mathcal{N}}}_{z}{\partial }_{y}\,{{\mathcal{N}}}_{x}\left(i{\omega }_{n}+\mu -{{\mathcal{N}}}_{0,{\bf{k}}+\frac{{\bf{q}}}{2}}\right){{\mathcal{N}}}_{y,{\bf{k}}-\frac{{\bf{q}}}{2}}\\  &  & \left.+{\partial }_{x}\,{{\mathcal{N}}}_{z}{\partial }_{y}\,{{\mathcal{N}}}_{x}\left(i{\omega }_{n}+i{\omega }_{m}+\mu -{{\mathcal{N}}}_{0,{\bf{k}}-\frac{{\bf{q}}}{2}}\right){{\mathcal{N}}}_{y,{\bf{k}}+\frac{{\bf{q}}}{2}}\right]\end{array}$$

and the denominator (${D}_{\alpha \beta }^{(z)}$) as

15 $${D}_{\alpha \beta }^{(z)}=\left[{\left(i{\omega }_{n}+\mu -{{\mathcal{N}}}_{0,{\bf{k}}+\frac{{\bf{q}}}{2}}\right)}^{2}-{| {\boldsymbol{\mathcal{N}}}_{{\bf{k}}+\frac{{\bf{q}}}{2}}| }^{2}\right]\times \left[{\left(i{\omega }_{n}+i{\omega }_{m}+\mu -{{\mathcal{N}}}_{0,{\bf{k}}-\frac{{\bf{q}}}{2}}\right)}^{2}-{| {\boldsymbol{\mathcal{N}}}_{{\bf{k}}-\frac{{\bf{q}}}{2}}| }^{2}\right]$$

a linear order expansion in **q** of the numerator gives

16 $${N}_{\alpha \beta }^{(z)}=-4i{e}^{2}\left[2{\rm{q}}\,{| {\boldsymbol{\mathcal{N}}}_{{\bf{k}}}| }^{3}({\nabla }_{{\bf{k}}}{{\mathcal{N}}}_{0,{\bf{k}}}\cdot {\Omega }_{\alpha }({\bf{k}}))+\,{\rm{q}}\,{\partial }_{x}{{\mathcal{N}}}_{z}{\partial }_{y}{{\mathcal{N}}}_{x}{\partial }_{z}{{\mathcal{N}}}_{y}(i{\omega }_{n}+\mu -{{\mathcal{N}}}_{0,{\bf{k}}})+2{({\boldsymbol{\mathcal{N}}}_{{\bf{k}}})}^{3}(i{\omega }_{m}){\Omega }_{z}({\bf{k}})\right]$$

where, Ω_*α*_(**k**) (*α* = *x*, *y*, *z*) is the Berry curvature given in Eq. (7). Since we are interested in the q-linear terms in Λ_*αβ*_, we calculate the Matsubara frequency sum for the first term in Eq. (16)

17 $$\begin{array}{ccc}{S}_{1} & = & \frac{1}{\beta }\sum _{n}\,\left[\frac{-8\,{\rm{q}}\,i{e}^{2}{| {\boldsymbol{\mathcal{N}}}_{{\bf{k}}}| }^{3}}{[{(i{\omega }_{n}+\mu -{{\mathcal{N}}}_{0,{\bf{k}}})}^{2}-{| {\boldsymbol{\mathcal{N}}}_{{\bf{k}}}| }^{2}]}\times \frac{{\nabla }_{{\bf{k}}}{{\mathcal{N}}}_{0,{\bf{k}}}\cdot {\Omega }_{\alpha }({\bf{k}})}{[{(i{\omega }_{n}+i{\omega }_{m}+\mu -{{\mathcal{N}}}_{0,{\bf{k}}})}^{2}-{| {\boldsymbol{\mathcal{N}}}_{{\bf{k}}}| }^{2}]}\right]\\  & = & -8i\,{\rm{q}}\,{e}^{2}{| {\boldsymbol{\mathcal{N}}}_{{\bf{k}}}| }^{3}\sum _{l}\,\frac{{(-1)}^{l}{n}_{F}({{\mathcal{E}}}_{l})({\nabla }_{{\bf{k}}}{{\mathcal{N}}}_{0,{\bf{k}}}\cdot {\Omega }_{\alpha }({\bf{k}}))}{| {\boldsymbol{\mathcal{N}}}_{{\bf{k}}}| [\left(i\right.{({\omega }_{m})}^{2}-4{| {\boldsymbol{\mathcal{N}}}_{{\bf{k}}}| }^{2}]}\end{array}$$

for the q-linear part of the second term in Eq. (16), we evaluate the sum

18 $${S}_{2}=\frac{1}{\beta }\sum _{n}\,\left[\frac{-4\,{\rm{q}}\,i{e}^{2}{\partial }_{x}{{\mathcal{N}}}_{z}{\partial }_{y}{{\mathcal{N}}}_{x}{\partial }_{z}{{\mathcal{N}}}_{y}}{\left[{(i{\omega }_{n}+\mu -{{\mathcal{N}}}_{0,{\bf{k}}})}^{2}-{| {\boldsymbol{\mathcal{N}}}_{{\bf{k}}}| }^{2}\right]}\times \frac{i{\omega }_{n}+\mu -{{\mathcal{N}}}_{0,{\bf{k}}}}{\left[{(i{\omega }_{n}+i{\omega }_{m}+\mu -{{\mathcal{N}}}_{0,{\bf{k}}})}^{2}-{| {\boldsymbol{\mathcal{N}}}_{{\bf{k}}}| }^{2}\right]}\right].$$

Carrying out the summation, we find that the sum vanishes completely i.e. *S*_2_ = 0. Hence there is no contribution from this term.

Lastly, for the third term in Eq. (16), we need to calculate the Matsubara sum

19 $${S}_{3}=\frac{1}{\beta }\sum _{n}\,\left[\frac{-8i{e}^{2}{| {\boldsymbol{\mathcal{N}}}_{{\bf{k}}}| }^{3}}{{\left(i{\omega }_{n}+\mu -{{\mathcal{N}}}_{0,{\bf{k}}+\frac{{\bf{q}}}{2}}\right)}^{2}-{| {\boldsymbol{\mathcal{N}}}_{{\bf{k}}+\frac{{\bf{q}}}{2}}| }^{2}}\times \frac{(i{\omega }_{m}){\Omega }_{z}({\bf{k}})}{{\left(i{\omega }_{n}+i{\omega }_{m}+\mu -{{\mathcal{N}}}_{0,{\bf{k}}-\frac{{\bf{q}}}{2}}\right)}^{2}-{| {\boldsymbol{\mathcal{N}}}_{{\bf{k}}-\frac{{\bf{q}}}{2}}| }^{2}}\right]$$

we carry out the frequency sum and Taylor expansion to keep only the q-linear terms in *S*_3_, and finally, after doing the analytical continuation, the total contribution to the real part of the complex dynamic chiral magnetic conductivity along the *z*-direction can be expressed as

20 $$\,{\rm{Re}}\,\left[{\sigma }_{z}^{chiral}(\omega )\right]=4{e}^{2}\mathop{\sum }\limits_{l=1}^{2}{\int }_{{\bf{k}}}{| {\boldsymbol{\mathcal{N}}}_{{\bf{k}}}| }^{3}\left[\frac{{n}_{F}^{{\prime} }({{\mathcal{E}}}_{l}){\Omega }_{z}({\bf{k}}){\partial }_{z}{{\mathcal{E}}}_{l}}{{\omega }^{2}-4{| {\boldsymbol{\mathcal{N}}}_{{\bf{k}}}| }^{2}}-\frac{{(-1)}^{l}{n}_{F}({{\mathcal{E}}}_{l})({\nabla }_{{\bf{k}}}{{\mathcal{N}}}_{0,{\bf{k}}}\cdot {\Omega }_{\alpha }({\bf{k}}))}{| {\boldsymbol{\mathcal{N}}}_{{\bf{k}}}| ({\omega }^{2}-4{\left|{\boldsymbol{\mathcal{N}}}_{{\bf{k}}}\right|}^{2})}\right]$$

which gives the Berry curvature-induced DCMC along the direction perpendicular to the tilt of the energy spectrum. In our work, we have only considered the Berry curvature-induced chiral magnetic conductivity. DCMC can also arise due to dynamic Zeeman coupling. However, the contribution of the dynamic Zeeman coupling is generally much smaller than that due to Berry curvature^19^.

### DCMC parallel to the tilt in the spectrum

When the electromagnetic wave is incident along the *x*-direction i.e. **q** = (q, 0, 0), we evaluate the *yz* and *zy*-components of Λ_*αβ*_. Writing Λ_*αβ*_ as

21 $${\Lambda }_{\alpha \beta }^{(x)}=\frac{{N}_{\alpha \beta }^{(x)}}{{D}_{\alpha \beta }^{(x)}}$$

and calculating the trace in Eq. (9), the numerator (${N}_{\alpha \beta }^{(x)}$) has the following form

22 $$\begin{array}{rcl}{N}_{\alpha \beta }^{(x)} & = & -4i{e}^{2}\left[{\partial }_{y}\,{{\mathcal{N}}}_{0}{\partial }_{z}\,{{\mathcal{N}}}_{z}\times \left({{\mathcal{N}}}_{x,{\bf{k}}+\frac{{\bf{q}}}{2}}\,{{\mathcal{N}}}_{y,{\bf{k}}-\frac{{\bf{q}}}{2}}-{{\mathcal{N}}}_{y,{\bf{k}}+\frac{{\bf{q}}}{2}}\,{{\mathcal{N}}}_{x,{\bf{k}}-\frac{{\bf{q}}}{2}}\right)\right.\\  &  & +\,{\partial }_{y}\,{{\mathcal{N}}}_{0}{\partial }_{z}\,{{\mathcal{N}}}_{y}\left({{\mathcal{N}}}_{z,{\bf{k}}+\frac{{\bf{q}}}{2}}\,{{\mathcal{N}}}_{x,{\bf{k}}-\frac{{\bf{q}}}{2}}-{{\mathcal{N}}}_{x,{\bf{k}}+\frac{{\bf{q}}}{2}}\,{{\mathcal{N}}}_{z,{\bf{k}}-\frac{{\bf{q}}}{2}}\right)\\  &  & +\,{\partial }_{y}\,{{\mathcal{N}}}_{0}{\partial }_{z}\,{{\mathcal{N}}}_{x}\left({{\mathcal{N}}}_{y,{\bf{k}}+\frac{{\bf{q}}}{2}}\,{{\mathcal{N}}}_{z,{\bf{k}}-\frac{{\bf{q}}}{2}}-{{\mathcal{N}}}_{z,{\bf{k}}+\frac{{\bf{q}}}{2}}\,{{\mathcal{N}}}_{y,{\bf{k}}-\frac{{\bf{q}}}{2}}\right)\\  &  & +\,{\partial }_{z}\,{{\mathcal{N}}}_{0}{\partial }_{y}\,{{\mathcal{N}}}_{x}\left({{\mathcal{N}}}_{z,{\bf{k}}+\frac{{\bf{q}}}{2}}\,{{\mathcal{N}}}_{y,{\bf{k}}-\frac{{\bf{q}}}{2}}-{{\mathcal{N}}}_{y,{\bf{k}}+\frac{{\bf{q}}}{2}}\,{{\mathcal{N}}}_{z,{\bf{k}}-\frac{{\bf{q}}}{2}}\right)\\  &  & +\,{\partial }_{y}\,{{\mathcal{N}}}_{x}{\partial }_{z}\,{{\mathcal{N}}}_{z}\left(i{\omega }_{n}+\mu -{{\mathcal{N}}}_{0,{\bf{k}}+\frac{{\bf{q}}}{2}}\right){{\mathcal{N}}}_{y,{\bf{k}}-\frac{{\bf{q}}}{2}}\\  &  & -\,{\partial }_{y}\,{{\mathcal{N}}}_{x}{\partial }_{z}\,{{\mathcal{N}}}_{y}\left(i{\omega }_{n}+\mu -{{\mathcal{N}}}_{0,{\bf{k}}+\frac{{\bf{q}}}{2}}\right){{\mathcal{N}}}_{z,{\bf{k}}-\frac{{\bf{q}}}{2}}\\  &  & +\,{\partial }_{y}\,{{\mathcal{N}}}_{x}{\partial }_{z}\,{{\mathcal{N}}}_{y}\left(i{\omega }_{n}+i{\omega }_{m}+\mu -{{\mathcal{N}}}_{0,{\bf{k}}-\frac{{\bf{q}}}{2}}\right){{\mathcal{N}}}_{z,{\bf{k}}+\frac{{\bf{q}}}{2}}\\  &  & \left.-\,{\partial }_{y}\,{{\mathcal{N}}}_{x}{\partial }_{z}\,{{\mathcal{N}}}_{z}\left(i{\omega }_{n}+i{\omega }_{m}+\mu -{{\mathcal{N}}}_{0,{\bf{k}}-\frac{{\bf{q}}}{2}}\right){{\mathcal{N}}}_{y,{\bf{k}}+\frac{{\bf{q}}}{2}}\right]\end{array}$$

and the denominator (*D*_*αβ*_) will be

23 $${D}_{\alpha \beta }^{(x)}=\left[{\left(i{\omega }_{n}+\mu -{{\mathcal{N}}}_{0,{\bf{k}}+\frac{{\bf{q}}}{2}}\right)}^{2}-{| {\boldsymbol{\mathcal{N}}}_{{\bf{k}}+\frac{{\bf{q}}}{2}}| }^{2}\right]\times \left[{\left(i{\omega }_{n}+i{\omega }_{m}+\mu -{{\mathcal{N}}}_{0,{\bf{k}}-\frac{{\bf{q}}}{2}}\right)}^{2}-{| {\boldsymbol{\mathcal{N}}}_{{\bf{k}}-\frac{{\bf{q}}}{2}}| }^{2}\right].$$

A linear order expansion of the numerator in **q** gives

24 $${N}_{\alpha \beta }^{(x)}=-\,4i{e}^{2}\left[2{\rm{q}}\,{| {\boldsymbol{\mathcal{N}}}_{{\bf{k}}}| }^{3}({\nabla }_{{\bf{k}}}{{\mathcal{N}}}_{0,{\bf{k}}}\cdot {\Omega }_{\alpha }({\bf{k}}))+\,{\rm{q}}\,{\partial }_{x}{{\mathcal{N}}}_{z}{\partial }_{y}{{\mathcal{N}}}_{x}{\partial }_{z}{{\mathcal{N}}}_{y}(i{\omega }_{n}+\mu -{{\mathcal{N}}}_{0,{\bf{k}}})+2{| {\boldsymbol{\mathcal{N}}}_{{\bf{k}}}| }^{3}(i{\omega }_{m}){\Omega }_{x}({\bf{k}})\right]$$

where, Ω_*α*_(**k**) (*α* = *x*, *y*, *z*) is the Berry curvature given in Eq. (7).

After performing the Matsubara sum and analytical continuation, similar to the previous section, we obtain the real part of ${\sigma }_{x}^{chiral}(\omega )$ as

25 $$\begin{array}{ccc}\,{\rm{Re}}\,\left[{\sigma }_{x}^{chiral}(\omega )\right] & = & 4{e}^{2}\mathop{\sum }\limits_{l=1}^{2}{\int }_{{\bf{k}}}{| {{\mathcal{N}}}_{{\bf{k}}}| }^{3}\left[\frac{{n{\prime} }_{F}({{\mathcal{E}}}_{l}){\Omega }_{x}({\bf{k}}){\partial }_{x}{{\mathcal{E}}}_{l}}{{\omega }^{2}-4{| {\boldsymbol{\mathcal{N}}}_{{\bf{k}}}| }^{2}}-\frac{{(-1)}^{l}{n}_{F}({{\mathcal{E}}}_{l})({\nabla }_{{\bf{k}}}{{\mathcal{N}}}_{0,{\bf{k}}}\cdot {\Omega }_{\alpha }({\bf{k}}))}{| {\boldsymbol{\mathcal{N}}}_{{\bf{k}}}| ({\omega }^{2}-4{| {\boldsymbol{\mathcal{N}}}_{{\bf{k}}}| }^{2})}\right.\\  &  & \left.+\frac{{(-1)}^{l}{n}_{F}({{\mathcal{E}}}_{l}){\omega }^{2}}{| {\boldsymbol{\mathcal{N}}}_{{\bf{k}}}| {({\omega }^{2}-4{| {\boldsymbol{\mathcal{N}}}_{{\bf{k}}}| }^{2})}^{2}}2{\Omega }_{x}({\bf{k}}){\partial }_{x}{{\mathcal{N}}}_{0,{\bf{k}}}\right]\end{array}$$

## Anisotropy in Rotary Power

In an inversion-symmetry-breaking material with non-zero chiral magnetic conductivity, electromagnetic waves with left and right circular polarizations possess different velocities^35,36^. This is known as optical activity, which leads to the rotation of the plane of polarization of the transmitted wave (per unit length *L*). This rotary power is a characteristic feature of a material which breaks the inversion symmetry and has a finite chiral magnetic conductivity^48,49^. The real part of the chiral magnetic conductivity can be obtained from the rotary power (${\mathcal{R}}$) using the following relation:

26 $${\mathcal{R}}=\frac{d\theta }{dL}=\frac{h}{2c{e}^{2}}\,{\rm{Re}}\,[{\sigma }^{chiral}(\omega )]$$

where, *c* is the speed of light in vacuum.

**Figure 2:**
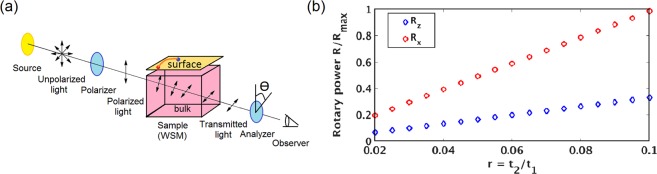
(**a**) Experimental arrangement to measure the rotary power. (**b**) Rotary power as a function of the tilt parameter *r* = $\frac{{t}_{2}}{{t}_{1}}$ for type-II WSMs for a fixed energy of the incident beam (E_*em*_ = 50 meV). We use *k*_0_ = $\frac{\pi }{4}$, *δ* = 2 and chemical potential *μ* = 20 meV. The blue curve shows the angle of rotation along the *z*-direction and the red curve is the rotary power along the *x*-direction. Clearly, as the tilt parameter increases, the anisotropy in the rotary power increases remarkably.

For estimating the value of the chiral magnetic conductivity, we use the infrared photons of energy 50 meV < E_*em*_(*ℏω*) < 1.7 eV because infrared frequencies are suitable for experimental measurement of rotary power. The experimental arrangement for detecting the optical activity is shown in Fig. 2(a).

At very low temperature (T → 0), ${n{\prime} }_{F}({{\mathcal{E}}}_{l})$ reduces to a delta function and as a result the main contribution to *σ*^*chiral*^(*ω*) comes from such terms in the expressions for DCMC (Eqs. (20) and (25)) which are proportional to the derivative of the Fermi function. We have numerically calculated the rotary powers ${{\mathcal{R}}}_{z}$ and ${{\mathcal{R}}}_{x}$ when light is incident along the *z* and *x* direction respectively.

Moreover, the calculations of rotary power for the two incident directions show that the rotation of the incident beam in the *x*-direction is almost three times that in the *z*-direction i.e., the rotary power is larger in the direction of the tilt of the Weyl point. It can be understood from Eqs. (20) and (25), which show that the dynamic chiral magnetic conductivity along a particular direction is proportional to the derivative of the energy spectrum ${\partial }_{\alpha }{{\mathcal{E}}}_{l}$ where *α* = *z*, *x*. Using Eq. (5), we write

27 $${\partial }_{\alpha }{{\mathcal{E}}}_{l}={\partial }_{\alpha }{{\mathcal{N}}}_{0,{\bf{k}}}+{(-1)}^{l}{\partial }_{\alpha }| {\boldsymbol{\mathcal{N}}}_{{\bf{k}}}| $$

and from Eq. (4), we get

$${\partial }_{z}{{\mathcal{E}}}_{l}={(-1)}^{l}{{t}_{1}}^{2}\sin {k}_{z}\left[\frac{\cos {k}_{z}+\delta [2\delta (1-\cos {k}_{z})+(\cos {k}_{0}-\cos {k}_{x})+(\cos {k}_{0}-\cos {k}_{y})]}{| {\boldsymbol{\mathcal{N}}}_{{\bf{k}}}| }\right]$$

and

28 $${\partial }_{x}{{\mathcal{E}}}_{l}=-{t}_{2}\left[\sin ({k}_{x}+{k}_{y})+\delta \ \sin ({k}_{x}-{k}_{y})\right]+{(-1)}^{l}{{t}_{1}}^{2}\sin {k}_{x}\left[\frac{(\cos {k}_{0}-\cos {k}_{x})+\delta (1-\cos {k}_{z})}{| {\boldsymbol{\mathcal{N}}}_{{\bf{k}}}| }\right].$$

The contribution of the ${\partial }_{\alpha }| {\boldsymbol{\mathcal{N}}}_{{\bf{k}}}|$ term is almost equal for both the directions. However, the ${\partial }_{\alpha }{{\mathcal{N}}}_{0,{\bf{k}}}$ term is zero for the *z*-direction while for the *x*-direction, it is non-zero and proportional to the tilt parameter (*r*), which is much larger than the ${\partial }_{\alpha }| {\boldsymbol{\mathcal{N}}}_{{\bf{k}}}|$ term. Since the angle of rotation of the transmitted beam depends on the real part of the chiral magnetic conductivity (Eq. (26)), which is proportional to the derivative of ${{\mathcal{E}}}_{l}$, rotary power will enhance along the tilt direction.

As seen from Fig. 2(b), the rotary power increases rapidly along the *x*-direction with the increase in the tilt parameter, whereas, the change in the rotary power is much smaller along the *z*-direction. Thus the anisotropic property of the optical activity of the transmitted beam can be a characteristic feature of an inversion-symmetry-broken tilted WSM. Also, Fig. 2(b) indicates that if electromagnetic waves are shone on a tilted WSM, the rotary power will be maximum when the incident beam falls along the direction of the tilt i.e, the direction in which the rotary power is maximum will determine the tilt direction of the Weyl semimetal. This result has an immense experimental importance since by simply tuning the direction of the incident wave and measuring the rotary power one can determine the tilt direction of an inversion-broken tilted WSM.

## Continuum Model

Expanding the lattice Hamiltonian around the *i*th Weyl point, we construct a low energy linearized Hamiltonian:

29 $$\begin{array}{rcl}H({\bf{k}}) & = & [{\Delta }_{i}-({k}_{x}-{K}_{x,i}){\gamma }_{x,i}-({k}_{y}-{K}_{y,i}){\gamma }_{y,i}]{\sigma }_{0}\\  &  & +[{t}_{1}(\cos {k}_{0}-{K}_{x,i})+({k}_{x}-{K}_{x,i}){v}_{x,i}]{\sigma }_{z}\\  &  & +\,[{t}_{1}(\cos {k}_{0}-{K}_{y,i})+({k}_{y}-{K}_{y,i}){v}_{y,i}]{\sigma }_{x}+{t}_{1}({k}_{z}-{K}_{z,i}){\sigma }_{y}\end{array}$$

where *K*_*α*,*i*_ is the *α*(=*x*, *y*)-th co-ordinate of the *i*(=1 − 4)-th Weyl node at the *k*_*z*_ = 0 plane and ${\Delta }_{i}={t}_{2}[\cos ({K}_{x,i}+{K}_{y,i})+\delta \cos ({K}_{x,i}-{K}_{y,i})]$ is the energy position of the *i*th Weyl point. The velocity components are given by

30 $$\begin{array}{ccc}| {\gamma }_{x,i}|  & = & | {t}_{2}[\sin ({K}_{x,i}+{K}_{y,i})+\delta \sin ({K}_{x,i}-{K}_{y,i})]| /\hslash \\ | {\gamma }_{y,i}|  & = & | {t}_{2}[\sin ({K}_{x,i}+{K}_{y,i})-\delta \sin ({K}_{x,i}-{K}_{y,i})]| /\hslash \\ | {v}_{x,i}|  & = & \frac{| {t}_{1}\sin {k}_{x,i}| }{\hslash }\quad \,{\rm{and}}\,\quad | {v}_{y,i}| =\frac{| {t}_{1}\sin {k}_{y,i}| }{\hslash }.\end{array}$$

In the continuum model, we calculate the real parts of the dynamical chiral magnetic conductivities ${\sigma }_{z}^{chiral}(\omega )$ and ${\sigma }_{x}^{chiral}(\omega )$ in the zero temperature limit. Since the right-handed and left-handed Weyl points are located at ±(*k*_0_, *k*_0_, 0) and ±(*k*_0_, −*k*_0_, 0) respectively, for a fixed ${k}_{0}=\frac{\pi }{4}$, the dynamic chiral magnetic conductivities in the continuum model become

31 $$\,{\rm{Re}}\,\left[{\sigma }_{z}^{chiral}(\omega )\right]={(\frac{3}{2})}^{\frac{3}{2}}\frac{{e}^{2}}{4{\pi }^{2}}\left[\frac{{\mu }_{{R}^{3}}}{{\omega }^{2}-4{\mu }_{{R}^{2}}}+\frac{{\mu }_{{L}^{3}}}{{\omega }^{2}-4{\mu }_{{L}^{2}}}\right]$$

32 $$\,{\rm{Re}}\,\left[{\sigma }_{x}^{chiral}(\omega )\right]={(\frac{3}{2})}^{\frac{3}{2}}\frac{{e}^{2}}{\sqrt{2}{\pi }^{2}}\left[(-\frac{{t}_{2}}{{t}_{1}}+\frac{1}{2\sqrt{2}})\frac{{\mu }_{{R}^{3}}}{{\omega }^{2}-4{\mu }_{{R}^{2}}}+(-\frac{{t}_{2}\delta }{{t}_{1}}+\frac{1}{2\sqrt{2}})\frac{{\mu }_{{L}^{3}}}{{\omega }^{2}-4{\mu }_{{L}^{2}}}\right]$$

where *μ*_*R*_ and *μ*_*L*_, the effective chemical potentials of the right-handed and left-handed Weyl points respectively, are given by

33 $${\mu }_{R}=\frac{\mu -{t}_{2}\delta }{1-\sqrt{2}\frac{{t}_{2}}{{t}_{1}}}\quad \,{\rm{and}}\,\quad {\mu }_{L}=\frac{\mu -{t}_{2}}{1-\sqrt{2}\frac{{t}_{2}\delta }{{t}_{1}}}$$

with *μ* being the conventional chemical potential.

**Figure 3:**
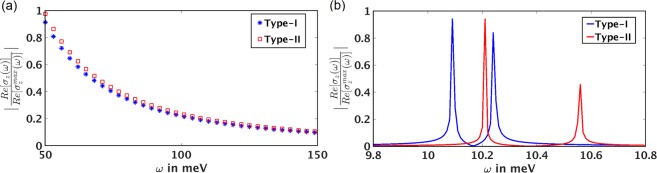
Frequency dependence of the real part of ${\sigma }_{z}^{chiral}(\omega )$ obtained from the continuum model: (**a**) in the high frequency regime, it follows the universal $\frac{1}{{\omega }^{2}}$ decay for both type-I and type-II WSMs. (**b**) When the energy of the incident electromagnetic wave is twice the effective chemical potentials of the left-handed and right-handed Weyl nodes, Re[${\sigma }_{z}^{chiral}(\omega )$] displays sharp peaks. For type-I WSMs, the peaks are close to each other and are situated at the low frequency part of the electromagnetic spectrum. For the type-II case, the position of the peaks are shifted to the higher frequency part of the spectrum and the frequency separation of the peaks increases. We choose *δ* = 2, *μ* = 5 meV, *t*_1_ = 1 meV, *t*_2_ = 0.02 meV and 0.009 meV for type-I and type-II limits respectively.
(ii) When the energy of the incident electromagnetic wave is twice the effective chemical potentials of the right-handed and left-handed Weyl nodes the dynamic chiral magnetic conductivity displays sharp peaks. As seen from Fig. 3(b), the real part of ${\sigma }_{z}^{chiral}(\omega )$ shows two peaks at 2*μ*_*R*_ and 2*μ*_*L*_. Since *μ*_*R*_ and *μ*_*L*_ depend on the tilt parameter of the Weyl points, the positions of the low energy excitations can be varied by varying the tilt parameter *r*. This allows one to distinguish between the type-I and type-II WSMs. For type-I WSMs, the peaks are close to each other and are situated at the low frequency part of the spectrum. In the type-II limit, the tilting of the Weyl nodes increases. As a result, the positions of the peaks are shifted to higher frequency part of the spectrum and the frequency separation of the peaks increases, allowing one to distinguish between the type-I and type-II WSMs. Re$[{\sigma }_{x}^{chiral}(\omega )]$ shows similar dependence on *ω* in both the low and high frequency regimes.

From the continuum model with finite chiral chemical potential, we find:

(i) In the high frequency regime i.e. at frequencies much larger than the scattering rate and the effective chemical potentials of the Weyl points, DCMC follows the universal $\frac{1}{{\omega }^{2}}$ decay for both type-I and type-II WSMs, as shown in Fig. 3(a).

This result is in contrast to the behaviour of the Weyl semimetals with no tilt (type-I WSM), where the positions of the low energy peaks in the dynamic chiral conductivity are fixed at twice the effective chemical potentials of the Weyl nodes^19^.

## Summary and Conclusion

In conclusion, we use a lattice Hamiltonian for an inversion-asymmetric tilted Weyl semimetal with finite chiral chemical potential to calculate the Berry curvature-induced DCMC for two different directions of the incident electromagnetic wave. From the relation between the real part of DCMC and the optical activity, we show that DCMC can be experimentally detected by measuring the angle of rotation of the plane of polarization of the transmitted electromagnetic beam. It is found that an inversion broken tilted Weyl semimetal shows remarkable anisotropy in the optical activity and produces larger rotation in the direction of tilt of the energy spectrum, which could then be regarded as a characteristic feature of an inversion-asymmetric tilted WSM. This effect has a considerable experimental importance as by simply tuning the direction of the incident electromagnetic wave and measuring the rotary power, one can determine the tilt direction of an inversion-broken tilted WSM.

We have shown the frequency dependence of the dynamic chiral magnetic conductivity from a continuum model and calculated analytical expressions for the real part of DCMC. When the frequency of the incident electromagnetic wave is much larger than the scattering rate and the effective chemical potentials of the left-handed and right-handed Weyl points, DCMC is found to decrease as $\frac{1}{{\omega }^{2}}$, while in the low frequency regime, the real part of DCMC shows sharp peaks at twice the effective chemical potentials of the Weyl nodes. In the model under study, the effective chemical potentials of the left-handed and right-handed Weyl points are tilt-dependent. The shift of the positions of the low energy peaks can serve in this case as a distinguishing feature for type-I and type-II Weyl semimetals.

In the present work, we focus on the real part of the dynamic chiral magnetic conductivity (DCMC) as the real part of DCMC is related to the natural optical activity or rotary power, which can be experimentally observed as a topological magneto-electric effect of inversion-broken tilted Weyl semimetals. However, we would like to point out that the imaginary part of DCMC also shows some interesting features of a noncentrosymmetric tilted Weyl semimetal. The imaginary part is related to the absorptive part of the dynamic chiral magnetic conductivity. As shown by Mukherjee and Carbotte^50^, there is a range of energy of the incident electromagnetic wave for which both the positive or negative chiral Weyl nodes contribute to the absorptive part, whereas, for some other photon energy ranges, the chiral nodes contribute separately. Also, by tuning the chiral chemical potential, it is possible to find regions of the electromagnetic wave spectrum, where there is no absorption associated with the imaginary part of the dynamic chiral magnetic conductivity^50^.
